# A Pilot Study to Evaluate the Feasibility and Acceptability of a Tailored Multicomponent Rehabilitation Program for Adolescent and Young Adult (AYA) Cancer Survivors

**DOI:** 10.3390/cancers17071066

**Published:** 2025-03-22

**Authors:** Lauren Corke, David M. Langelier, Abha A. Gupta, Scott Capozza, Eric Antonen, Gabrielle Trepanier, Lisa Avery, Christian Lopez, Beth Edwards, Jennifer M. Jones

**Affiliations:** 1 Cancer Rehabilitation and Survivorship Program, Princess Margaret Cancer Centre, Toronto, ON M5G 2C4, Canada; lauren.corke@uhn.ca (L.C.); eric.antonen2@uhn.ca (E.A.); christian.lopez@uhn.ca (C.L.); beth.edwards@uhn.ca (B.E.); 2 Institute of Medical Sciences, University of Toronto, Toronto, ON M5S3K3, Canada; 3 Department of Clinical Neurosciences, Division on PM&R, University of Calgary, Calgary, AB T2N 1N4, Canada; david.langelier@albertahealthservices.ca; 4 Divison of Hematology/Oncology, The Hospital for Sick Children, Toronto, ON M5G 1E8, Canada; abha.gupta@sickkids.ca; 5 Adolescent and Young Adult Oncology Program, Princess Margaret Cancer Centre, Toronto, ON M5G 2C4, Canada; 6 Yale New Haven Health Outpatient Oncology Rehabilitation Services, Smilow Cancer Hospital Adult Cancer Survivorship Clinic, New Haven, CT 06519, USA; scott.capozza@ynhh.org; 7 Temerty Faculty of Medicine, University of Toronto, Toronto, ON M5S 3K3, Canada; g.trepanier@mail.utoronto.ca; 8 Department of Biostatistics, Princess Margaret Cancer Centre, Toronto, ON M5G 2C4, Canada; lisa.avery@uhn.ca

**Keywords:** adolescents, young adults, cancer rehabilitation, survivorship, self-management skills, exercise

## Abstract

Background: Adolescent and young adult (AYA) cancer survivors have unique needs, yet few tailored rehabilitation programs exist. CaRE-AYA is an 8-week multidimensional rehabilitation program for AYAs that targets impairments, activity limitations and participation restrictions and comprises an individualized exercise prescription and weekly group-based exercise and self-management education. Methods: We conducted a single-arm multi-method pragmatic feasibility study evaluating CaRE-AYA’s feasibility, acceptability, and safety (primary outcomes) and assessed disability, physical functioning, mental health, and social functioning at baseline (T0), immediately post-intervention (T1), and 3 months post-intervention (T2). Results: Twenty-five participants (median age: 32.8, 76% female) were enrolled. Participation (60%), retention (84% at T1, 72% at T2), and program adherence (76% for education classes, 74% for exercise classes) supported feasibility. Qualitative interviews (*n* = 9) supported strong perceptions of feasibility and acceptability. No serious adverse events (≥grade 3) attributed to CaRE-AYA occurred. Conclusions: The results suggest CaRE-AYA is feasible, acceptable, and safe. A randomized controlled trial is needed to determine the effectiveness.

## Simple Summary

Adolescent and young adult cancer survivors (AYAs) experience many unique challenges when facing a cancer diagnosis. Cancer rehabilitation in the AYA population could maximize function, activity and participation, and assist with the transition into survivorship. Despite this, the availability of cancer rehabilitation programs designed to address the specific needs of AYA survivors remains limited. The Cancer Rehabilitation and Exercise (CaRE-AYA) program is a multidimensional rehabilitation program tailored to the AYA population. The CaRE-AYA program includes an individualized exercise prescription, as well as 8 weeks of weekly group-based exercise and self-management skill education classes. This pilot study aimed to assess the feasibility, acceptability, and safety of the CaRE-AYA program. The CaRE-AYA program achieved a variety of a priori targets and was confirmed to be feasible, acceptable, and safe. The results of this study provide insight into the design of cancer rehabilitation programming for AYA survivors and provide a foundation for further adaptation and large-scale testing.

## 1. Introduction

Each year in Canada, more than 8000 adolescents and young adults (AYA) (15–39 years) are diagnosed with cancer [1]. This phase of life is vulnerable to many social, developmental, physical, and career changes [2,3,4,5,6], and a cancer diagnosis at this time can create many unique challenges for AYAs. This includes disruptions to major life milestones such as their education and careers, formation of intimate relationships, financial independence, and family development [2,3,4,6].

AYAs with cancer experience a higher 5-year survival rate than the general cancer population (86% and 65%, respectively) [7]. This, in combination with their young age, results in a large proportion of AYA cancer patients becoming long-term survivors [5]. AYA cancer survivors face several significant physical and psychosocial adverse effects of treatment [8,9,10,11,12,13,14] and many cancer treatments are known to cause impairment in one or more functional domains including mobility, cognition, independent living, and self-care [15]. In a study of 575 AYA Canadian cancer survivors aged 18–34, 88% of participants reported experiencing at least one physical concern [16]. The most frequently reported physical concerns included fatigue (78%), hormonal or fertility concerns (50%), and changes to concentration and memory (49%) [16]. These effects can persist many years following treatment and can lead to functional limitations that can diminish AYA survivor’s function and ability to participate fully in work and life roles [9,10,13], reduce overall quality of life (QoL) [17], and result in a higher prevalence of disability compared to their peers [18,19]. Survivors of AYA cancer also have a greater prevalence of comorbidities later in life than those without a history of cancer [11]. As a result, greater consideration of the management of the adverse effects of cancer and its treatment has been emphasized to minimize dysfunction and maximize function for the growing number of AYA cancer survivors.

Rehabilitation is defined by the World Health Organization as a field that addresses “the impact of a health condition on a person’s everyday life, by optimizing their functioning and reducing the experience of disability” [20]. Cancer rehabilitation is a vital component of cancer care that aims to reduce impairments, activity limitations and participation restrictions of patients through preventative, restorative, and supportive interventions [21]. Due to the presence of multiple impairments in many cancer survivors, effective cancer rehabilitation often necessitates a collaborative multidisciplinary approach. As opposed to targeted rehabilitation programs, which are often focused on only one impairment, multicomponent programs offer an advantage due to their holistic approach [22]. Multidimensional programs may be favourable to address the wide scope of concerns faced by AYA survivors. This is supported by the research of Aagesen and colleagues (2023), which emphasized a need to increase multicomponent and biopsychosocial-focused cancer rehabilitation for AYAs [23] as well as the need for peer-to-peer connection [23,24].

There are a growing number of studies demonstrating the benefits of multidimensional rehabilitation interventions (i.e., interventions with two or more components such as exercise, physical therapy, psychosocial counseling, nutritional counseling, etc.) [23]. However, there is a paucity of research addressing the need for cancer rehabilitation programs tailored to the AYA population, which has resulted in the limited availability of accessible programs for these patients [23,25,26]. Further, most AYA-specific programs have focused on one dimension of rehabilitation, such as broad exercise-based interventions [23,26,27] and have not taken an impairment-driven approach [27,28,29]. While these interventions have been demonstrated to be feasible in this population [8,27,28,29], a more comprehensive multidimensional rehabilitation approach is needed to address the needs of AYA cancer survivors experiencing cancer-related impairments and related disability [23].

In response, we developed a tailored multidimensional rehabilitation program for AYA cancer survivors experiencing cancer-related impairments (CaRE-AYA). This multi-method feasibility study aimed to assess the feasibility, acceptability, and safety of CaRE-AYA and to obtain preliminary estimates of its impact.

## 2. Materials and Methods

### 2.1. Design

We conducted a single-arm pragmatic, multi-method feasibility study to assess (1) the feasibility, (2) acceptability, and (3) safety of the CaRE-AYA program [30,31]. The study was approved by the Research Ethics Board at the University Health Network (REB#17-5218) and followed the Consolidated Standards of Reporting Trials (CONSORT) guidelines for pilot and feasibility studies [32]

### 2.2. Setting

The CRS Program at Princess Margaret offers specialized, impairment-focused rehabilitation to patients during and after cancer treatment, aiming to reduce disability and enhance functional independence. Patients are referred to CRS by their oncologists or general practitioner when they are experiencing cancer-related impairments such as persistent fatigue, deconditioning, difficulties with activities of daily living, and musculoskeletal issues. They then undergo a comprehensive combined assessment by a physiatrist, as well as a physiotherapist or occupational therapist. This assessment is 1.5 h in duration and allows for a comprehensive picture of the patient’s medical history, current function, care-related impairments, and their physical and social environment. Following this assessment, a personalized care plan is developed that may include specialty follow-up consultations (e.g., physiotherapy, occupational therapy, nutrition, sexual health, etc.). Patients can also be referred to the Cancer Rehabilitation and Exercise (CaRE) program, delivered at the Centre for Health Wellness and Cancer Survivorship (ELLICSR). The CaRE@ELLICSR program is an existing evidence-based eight-week group-based multidimensional intervention that consists of a weekly in-person 1 h group-based exercise class and a 1.5 h self-management skills education class delivered by specialized instructors [33]. CaRE@ELLICSR was designed to target the International Classification of Functioning, Disability and Health (ICF) domains [34], including function, activity, and participation and to support individuals in adapting their health behaviours such as physical activity, nutrition, and the management of common adverse effects by providing effective strategies and interventions informed by behavioural change theory [35]. CaRE@ELLICSR integrates behaviour change techniques such as goal setting, monitoring of the behaviour, social support, and repetition.

### 2.3. Participants and Recruitment

Potential participants were identified for CaRE-AYA during their CRS initial comprehensive assessment. Given the pragmatic nature of this study, the inclusion criteria were designed to be broad and minimally restrictive, aiming to recruit a patient population that closely resembles the real-world population who would receive the intervention being studied [36,37]. Patients were eligible to participate in CaRE-AYA if they (1) had received treatment for a primary cancer diagnosis of any tumor type at Princess Margaret; (2) were ≥18 and ≤39 years of age; (3) were experiencing cancer-related impairments and moderate to high levels of self-reported or objective disability through assessment by the CRS clinicians; (4) received medical clearance from the CRS team to participate in exercise; (5) could understand written and spoken English due to program content. Patients were excluded if they had a planned surgery during the study duration. Patients were not excluded if they were currently receiving chemotherapy, hormone therapy, immunotherapies, or radiation treatment.

Patients who met inclusion criteria were provided with information about the program and those who were interested were referred to the CaRE intake coordinator and subsequently contacted to schedule their initial baseline assessment. Participation in the research study evaluating the feasibility of the program was optional. Patients registered in the program were contacted by the study team prior to the baseline assessment and asked to provide prospective written informed consent to use their program data for research purposes. If a participant did not consent, they could still participate in the CaRE-AYA program. During the consenting process, participants also had the option to consent to an individual qualitative interview 3 months post-program (T2).

### 2.4. Intervention

In order to meet the specific needs of the AYA population receiving care at CRS, the CaRE@ELLICSR program was adapted based on the results of a needs assessment with AYA cancer patients (*n* = 72), input from an expert advisory workgroup that included AYA oncology (nursing, physiotherapy/patient rep) and cancer rehabilitation (physiotherapy, occupational therapy, kinesiology), and CaRE class instructors. Program adaptations and modifications were made to the group-based exercise and self-management skill education class content, program delivery, and available resources and were documented using an adapted version of the FRAME [38], a framework for reporting adaptations and modifications to evidence-based interventions. Program adaptations are summarized in Supplemental File S1.

**Figure 1:**
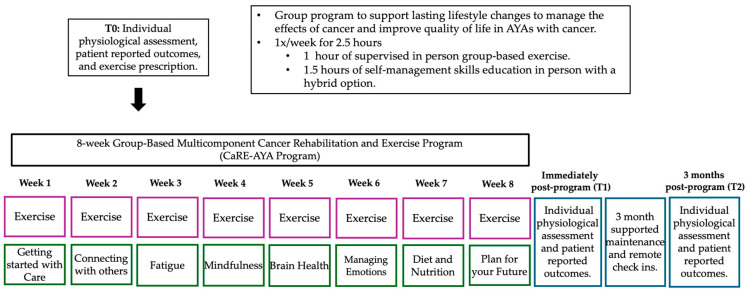
CaRE-AYA program timeline.

The resulting CaRE-AYA program is an 8-week tailored group program specific to AYA cancer patients delivered by the Princess Margaret CRS and AYA team (Figure 1). Effective cancer rehabilitation often necessitates a collaborative multidisciplinary approach [39]. The CaRE-AYA program included the following:

(1) Individualized exercise plan: Prior to the initiation of the 8-week CaRE-AYA program, each participant underwent an initial physiological assessment with a Registered Kinesiologist (RKin) (T0). Based on the initial assessment, the RKin prescribed an individualized exercise plan developed to correspond to the American College of Sports Medicine (ACSM) exercise guidelines for cancer survivors [40] and guided by the Cancer & Exercise Manual for Health and Fitness Professionals and the FITT Principle of Exercise Prescription [41]. The prescribed plan included moderate-intensity aerobic training at least three times a week for at least 30 min and resistance training at least twice weekly [40]. Exercise prescription was supported by a remote application (Physitrack™), a wearable activity tracker (Fitbit™), and the provision of resistance bands to support independent exercise in between the weekly group-based sessions. Physiological assessments were repeated immediately post-program (T1) and 3 months post-program (T2).

(2) Weekly group-based exercise classes: Each week, participants attended a one-hour in-person group-based exercise session. Exercise sessions were supervised by two RKins and began with a five-minute discussion on various exercise topics followed by a five-to-ten-minute circuit-style warmup. Following the warm-up, participants spent 30 min completing their individualized exercise plan, which was prescribed to them during their initial assessment by the RKin. Participants alternated between resistance exercises and two to three minutes of aerobic exercise with a machine or a warm-up station. Every other week, participants were introduced to an optional alternative exercise method (e.g., super setting, HIIT). These alternative exercise methods were added to the CaRE-AYA program based on the AYA’s interest. This weekly group-based exercise session allowed participants to ask questions, receive modifications, progress their exercise program, and communicate their goals with exercise professionals during the eight weeks. In addition to the weekly in-person sessions, participants were instructed to engage in independent exercise, consisting of both aerobic and resistance training, at least two to three times a week to meet their prescribed exercise dose.

(3) Weekly group-based self-management skills education classes: On the same day as the in-person exercise class, participants completed an in-person 1.5 h self-management skill education session delivered by Princess Margaret CRS staff. Self-management skill education classes were delivered by specialized instructors (e.g., occupational therapist, social worker, dietitian, neuropsychologist), and topics included (1) Getting Started with CaRE; (2) Cancer-related Fatigue; (3) Diet and Nutrition; (4) Managing Emotions; (5) Mindfulness; (6) Connecting with Others; (7) Brain Health; and (8) Plan for your Future.

(4) Supported maintenance phase: Following the 8-week in-person CaRE-AYA program, participants entered a supported maintenance phase. This included two monthly remote follow-up calls with the RKin (one month post-program and two months post-program). These follow-up calls were provided for continued guidance on the prescribed exercise plan and to support participants transitioning out of the CaRE-AYA program. No study data were collected during these follow-up calls. This supported maintenance phase concluded when participants attended their 3-month post-program assessment (T2) in person with the RKin.

### 2.5. Study Outcomes

Outcomes were defined and reported based on CONSORT guidelines and recommendations for pragmatic feasibility studies [31] and data were collected at three different timepoints, baseline (T0), immediately post-8-week intervention (T1), and 3 months post-intervention (T2). Additionally, a qualitative interview was conducted 3 months post-intervention (T2), with a subset of participants to assess the program’s acceptability.

Demographic and Clinical Data: Demographic and clinical information was obtained by chart review at baseline and was self-reported in the initial assessment questionnaire. Data included age, sex and gender, marital status, employment status, time since diagnosis, and primary cancer location.

Primary Outcomes: (1) Feasibility: The feasibility of the intervention was assessed by (1) participation rates; (2) retention rates; (3) program adherence; and (4) the frequency of missing outcomes from physiological assessments and patient-reported outcomes. In accordance with the Consolidated Standards of Reporting Trials (CONSORT) guidelines for pilot and feasibility studies [32], a priori targets were set using relevant literature [26,27,28,29,30,42,43,44,45,46]. The participation rate was calculated as the proportion of eligible participants who consented to the study compared to the overall number of eligible participants referred to the CaRE-AYA program (target ≥ 60%). Retention rates were calculated at two different timepoints (T1 and T2) (target ≥ 70% for T1 and T2)*,* and reasons for study dropout were documented. Program adherence was calculated as two separate rates, attendance to the self-management skill education sessions, and attendance to the exercise sessions (target ≥ 70%). Additionally, the frequency of missing outcomes for patient-reported outcomes and physiological assessments were reported. (2) Acceptability: The acceptability of the intervention and program methods was evaluated through in-depth semi-structured qualitative interviews. Interviews were conducted on Microsoft Teams with a subset of participants during the T2 assessment period. Each interview was approximately 30–60 min and comprised open-ended questions and relevant prompts addressing different program elements, barriers to program attendance, and suggested feedback for program improvement. (3) Safety: The safety of the CaRE-AYA program and study requirements were assessed throughout the study. Any adverse events arising from the CaRE-AYA program (e.g., exercise or self-management skills education classes) or study procedures (e.g., patient-reported outcomes and physiological assessments) were evaluated and scored on the CTCAE version 5.0. Participants were also instructed to inform the RKins if any adverse events occurred at home between the weekly sessions. All adverse events were documented, graded according to the CTCAE criteria, and evaluated to determine whether they were related to the intervention. Safety was confirmed if no intervention-related serious events, defined as grade > 3 on the CTCAEv5, occurred [46].

Secondary Outcomes: Participants underwent in-person physiological assessments with a trained exercise professional and completed a questionnaire package of patient-reported outcomes at baseline (T0), immediately post-program (T1), and 3 months post-program (T2). While the study was not powered to assess change, these assessments were conducted to assess their feasibility, acceptability, and safety and to provide pre- and post-descriptives and preliminary estimates to inform future research.

1. Disability was measured using the 12-item World Health Organization Disability Assessment Schedule 2.0 (WHO-DAS 2.0) [47]. Respondents rate their difficulty in engaging in particular activities on a scale from “none” (no difficulty) to “extreme or cannot do” on six domains of functioning. Scores range from 12 to 60, where higher scores indicate higher disability or loss of function [47].
2. Physical functioning was measured using the SF-36 physical functioning score and physical component summary [48]. Participants rate their difficulty in engaging in certain activities on a 6-point scale for each item. The physical functioning subscale includes 10 items. Scores are converted to a 0–100 range. A higher score indicates a more favourable health state [48]. The PCS includes measurements from the physical functioning, role-physical, bodily pain, and general health subscales.
3. Social functioning was measured using the Social Difficulties Inventory (SDI), which was developed for use in cancer care to evaluate different areas of potential difficulty in daily life [49]. Each item measures a different area of potential difficulty in daily life. Item scores are summed to calculate a total score. Scores greater than 10 indicate social distress [49].
4. Mood was measured using the Generalized Anxiety Disorder Questionnaire (GAD-7), which is an efficient tool in the screening and assessment of generalized anxiety disorder [50]. Question response options include “not at all”, “several days”, “more than half the days” and “nearly every day” scored as 0, 1, 2, and 3. Scores are summed to reveal a total score ranging between 0 and 21 with higher scores indicating higher symptomatology [50].
5. Activity levels were assessed using the Godin Sheppard Leisure Time Physical Activity Questionnaire (GSLTPAQ) [51]. This scale uses the metabolic equivalent of a task (METS) and is calculated by (Strenuous activity × 9 METS) + (moderate exercise × 5 METS) + (light exercise × 3 METS) = total leisure activity score [51]. Scores can then be categorized into insufficiently active (scores < 14), moderately active (scores 14–23), and active (scores > 23) [52].
6. Cardiorespiratory fitness was measured using the Six-minute Walk Test (6MWT), which is a cardiorespiratory test that measures the distance someone can walk in six minutes. Greater distances indicate a more favourable health state [40].
7. Grip strength was measured by the amount of force applied on a Jamar dynamometer. The amount of force from both hands was combined to create a total score. Increased force indicates a more favourable health state [53].

### 2.6. Data Analyses

Given the primary aim of this pilot study was to assess feasibility, acceptability, and safety, the sample size was not calculated using power analysis [31,54,55]. Many guidelines exist for recommended sample sizes of pilot studies, with suggested ranges of 10–35 participants per arm [56]. We aimed to run four CaRE-AYA groups with 6–12 consented participants over one year, resulting in a target sample size of *n* = 24–48. Having four different groups allowed for adaptations to the program between groups guided by participant feedback. Demographic and feasibility data were reported using descriptive statistics. Continuous data are reported as means and standard deviations along with median and range and categorical data are reported as frequencies and percentages. Interview transcripts were uploaded and analyzed on the research application “Dedoose” [57] and were coded by two independent coders (LC and EA). Braun and Clarke’s six phases of applied thematic analysis were used to identify themes and recognize patterns [58,59]. Thematic analysis was selected as it is a rigorous, yet flexible, method designed to identify themes transparently and credibly [58]. An initial codebook was developed deductively to align with the program’s main components (e.g., Exercise prescription, education classes, exercise classes) as well as acceptability categories (e.g., strengths, weaknesses). The transcripts were coded into these categories, and themes were identified to determine what the participants enjoyed or did not enjoy about the program and how it impacted their lives. Since Braun and Clarke’s applied thematic analysis is an iterative process [47], codes were also developed inductively throughout the analysis process. Additional codes were added to the codebook. Rigor was established by adhering closely to Braun and Clarke’s six phases of thematic analysis and practicing research reflexivity.

Secondary outcomes were analyzed using linear mixed-effect models. The number and proportion of missing observations were calculated at each timepoint. Mixed-effects linear models were fit to each outcome to estimate the longitudinal changes in outcomes from baseline to T1 and from T1 to T2 (fixed effects), allowing baseline values to vary by participant (random effect). A mixed-effects approach was chosen because it allows estimation of change over time using all the observed data for each participant and does not require imputation of outcomes. Furthermore, the model allows separate estimation of the longitudinal change (from baseline to T1 and from T1 to T2) while controlling for correlated outcomes over time within participants. Total change from baseline to end of follow-up (T0 to T2) was estimated for each outcome based on the model parameters. Standardised mean difference effect sizes were calculated for the total change from T0 to T2. All statistical analyses were conducted using SAS Version 9.3 or R version 3.5.1.

## 3. Results

From May 2023 to March 2024, four CaRE-AYA groups were piloted. A total of *n* = 25 AYA patients consented to participate in the study. Participant demographic and clinical characteristics are presented in Table 1. Variations resulting from logistical factors (e.g., weekday of the class sessions) or recruitment rates (e.g., the size of each group) existed between the four CaRE-AYA groups. Groups one and two both consisted of six participants, group three consisted of three participants, and group four contained eleven participants. However, other variations were a result of program adaptations that were implemented in response to participant feedback on evaluation forms and informal feedback from CRS class instructors. A summary of between-group adaptations using the A-FRAME is available in Supplemental File S2.

### 3.1. Feasibility

**Figure 2:**
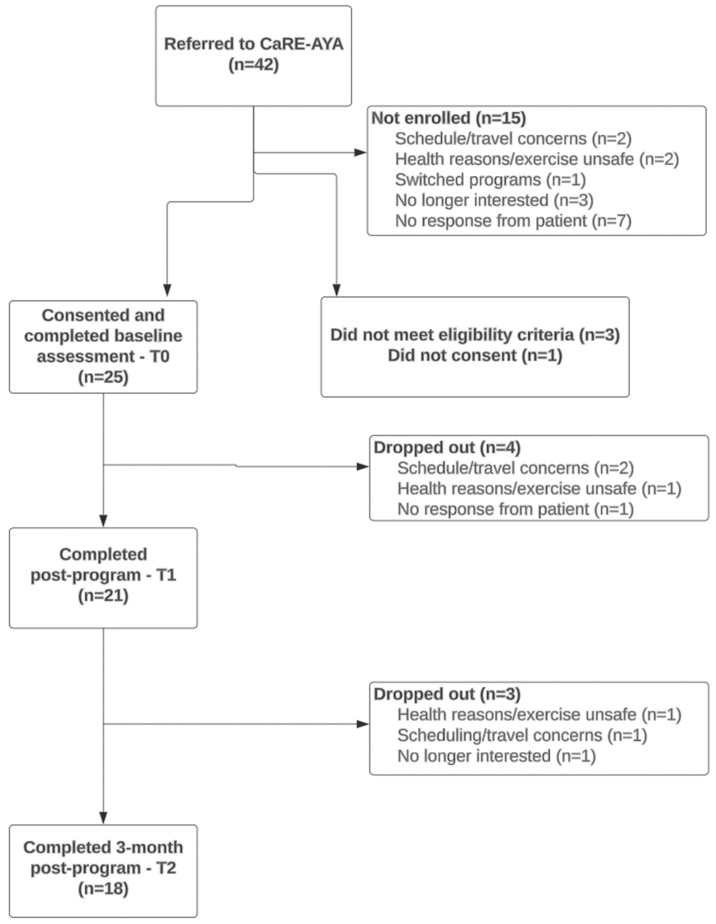
Adapted CONSORT participant flow diagram.

Figure 2 outlines an adapted CONSORT diagram for pilot feasibility studies that was utilized to track participant flow throughout the study [31]. Between March 2023 and January 2024, 42 patients were referred to the CaRE-AYA program and 28 patients were registered and participated in the 8-week CaRE-AYA program. Of those who participated in the program, 25 consented to the study, resulting in a participation rate of 60%. Three patients did not consent to the study, two did not meet the study eligibility criteria, and one patient did not provide consent. The retention rate at the T1 timepoint was 84% as 21 participants completed the 8-week program and the T1 assessments. Four participants dropped out of the study before T1. The retention rate at T2 was 72%, as 18 out of 25 included participants were retained at T2.

Class attendance rates were calculated based on the number of participants who remained in the study at T1 (*n* = 21). The adherence to the education and exercise classes was 76% and 74%, respectively (*n* = 21). The education classes with the highest attendance were “Getting Started with CaRE” (*n* = 20, 95%), “Connecting with Others” (*n* = 19, 90%), and “Mindfulness” (*n* = 18, 86%). “Cancer-related Fatigue” (*n* = 15, 71%) and “Plan for Your Future” (*n* = 14, 66%) had the lowest attendance.

### 3.2. Acceptability

#### 3.2.1. Qualitative Interviews

Overall, 19 participants consented to participate in a post-intervention qualitative interview. At first, a convenience sample of participants who had completed the 8-week program and 3-month follow-up and had consented to an interview were invited to participate. However, following several interviews, male participants and multiple participants from each CaRE-AYA group were purposively sampled to encompass diverse viewpoints and group experiences. Qualitative interviews were conducted with *n* = 9 participants, after which point no new information or themes regarding the feasibility and acceptability of the program emerged from the interview data. This suggested data saturation may have been achieved. Most interviewees had a hematological cancer diagnosis (*n* = 6, 67%) and were not on active treatment during the CaRE-AYA program (*n* = 8, 89%). This was reflective of the overall CaRE-AYA population as hematological cancer was the most common diagnosis among CaRE-AYA participants (*n* = 11, 44%), and the majority of CaRE-AYA participants were not on active treatment (*n* = 19, 76%). Additionally, in alignment with the overall CaRE-AYA population, the majority of interviewees were female (*n* = 7, 78%). The qualitative interviews successfully captured perspectives from a broad age range, including both the youngest (aged 20) and the oldest (aged 38) CaRE-AYA participants. Interview coding was completed by two independent coders using the research application Dedoose™ (version number: 9.0.107). Through the thematic analysis of interview transcripts, four major themes emerged from that data. A summary of these themes is found below; a more detailed description of each theme and sub-theme, along with example quotes, is available in Supplemental File S3.

##### Theme 1: Program Benefits Experienced by CaRE-AYA Participants

Participants shared that the CaRE-AYA program taught them new knowledge, skills, and habits to manage their cancer-related symptoms. As a result of this, some participants reported experiencing an improvement in their cancer-related symptoms and overall health. Participants emphasized the supportive nature of the CaRE-AYA program and appreciated the support they received from program staff and other group members. They also appreciated that the program was AYA-specific. They felt the program content was tailored to their needs and enjoyed being in a group with other AYA survivors.

##### Theme 2: Facilitators of the CaRE-AYA Program Benefits

Participants felt the program benefits were facilitated by the program design and access to helpful resources. Participants enjoyed the weekly exercise and self-management skills classes and felt the duration of these classes was appropriate. They also found the individualized exercise prescription and access to a remote application (Physitrack™) and a wearable activity tracker (FitBit™) very helpful. Additionally, participants had no concerns with the study requirements. They found the assessments to be appropriate, safe, and a reasonable length.

##### Theme 3: Identified Drawbacks of the CaRE-AYA Program

Varying information needs existed among the CaRE-AYA participants. Therefore, some participants did not find that all the content applied to their specific needs. Additionally, participants requested more interactivity in the self-management skill education classes. More specifically, participants wished for increased group discussion time.

##### Theme 4: Barriers to Program Attendance and Participation Faced by CaRE-AYA Participants

Participants from groups with early morning classes at 8:30 a.m. and 9:00 a.m. emphasized that these times were a barrier to attendance and that they would have preferred a later class time. Symptom burden, transportation, and illness unrelated to cancer posed additional barriers to program attendance. Additionally, participants suggested the program aims for increased visibility among AYAs, in particular younger AYAs.

### 3.3. Safety Outcomes

No serious adverse events (≥grade 3 on the CTCAE) related to the program occurred during the in-person self-management skills education classes, group-based exercise classes, or while exercising at home between the weekly sessions. Only one adverse event (CTCAE 1, mild), muscle soreness from exercise, was definitely attributed to the intervention. However, this was expected as muscle soreness is a typical response to increased exercise frequency [60].

### 3.4. Secondary Outcomes

**Figure 3:**
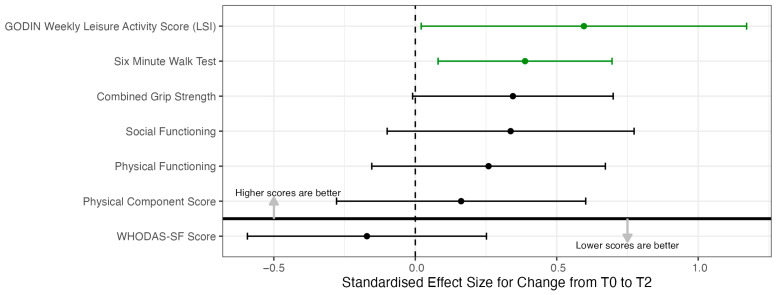
Standardised effect size from T0 to T2 across outcomes.

Exploratory analysis of secondary clinical outcomes was conducted to obtain estimates of the CaRE-AYA program’s effects on disability, physical functioning, mental health, and social functioning. Figure 3 plots all the standardised effect sizes for the outcomes for change from T0 to T2. The confidence intervals for the estimated change and estimated effect size are quite wide, indicating low precision. However, this was expected due to the small sample size of the study. The summary statistics over time by each outcome are available in Supplemental File S4.

## 4. Discussion

In response to the gaps in AYA cancer care and the call for tailored cancer rehabilitation in the AYA population, we adapted the CaRE@ELLICSR program for the AYA population (CaRE-AYA) and conducted a pilot study to examine the feasibility, acceptability, and safety of this new program. Overall, the results indicate that the newly developed CaRE-AYA program’s content, delivery, and evaluation methods were feasible and acceptable. The CaRE-AYA program was also determined to be safe as no serious adverse events resulting from the program procedures occurred. Study procedures, including patient-reported outcomes and physiological assessments, were also determined to be feasible, acceptable, and safe. The study provided important information and considerations for the design of future CaRE-AYA studies.

A priori objectives for each feasibility outcome, including the participation rate, retention rates, and program adherence, were met, demonstrating that the CaRE-AYA program and study procedures were feasible. The retention rate of 84% and adherence outcomes (76% and 74%) were higher than our targets and aligned with ranges reported in similar interventions [26,27,28]. The participation rate of 60% was met but not exceeded. To increase participation, using strategies suggested by relevant literature [27,61,62,63], we recommend that the physical benefits of the rehabilitation program be highlighted to patients before referral and that study advertisements be distributed in person at the cancer centre and virtually through social media and email. Future iterations of the CaRE-AYA program should also focus on increasing the recruitment of biological males, gender minorities, and younger AYAs (aged 18–25). Additionally, the CaRE-AYA program was delivered in person at a hospital located in the downtown core of a large metropolitan area. This may pose a barrier for participants who must travel from outside the city to attend or who do not desire to return to the hospital for a program. Additionally, the timing of the program, which was early morning for the first two groups, may also pose a challenge to participation, especially for those dealing with impairments such as cancer-related fatigue. Providing the option to complete home-based virtual cancer rehabilitation can increase access for those facing barriers to in-person programs [64], and scheduling AYA rehabilitation programming later in the morning or afternoon can further increase accessibility.

Feedback from the semi-structured qualitative interviews suggested high acceptability of the CaRE-AYA program among AYA participants. The program design and study procedures were acceptable for participants, with participants reporting satisfaction with the 8-week program length, in-person format with a hybrid option, duration of the classes, and study assessments. Participants described experiencing a wide range of benefits from the CaRE-AYA program and stressed the importance of an AYA-specific program. They greatly appreciated the opportunity provided by the CaRE-AYA program to socialize and learn from peers with similar experiences. This observation aligns with previous findings in the literature, suggesting AYA cancer survivors would benefit from interaction with other AYA cancer survivors [65]. Participants who did not already own a wearable activity tracker were equipped with a FitBit™ device to facilitate self-monitoring of exercise behaviours and outcomes. Participants suggested that the wearable activity trackers helped promote accountability, supporting their use in exercise-based AYA cancer rehabilitation programming. Based on participant feedback, minor content changes and increased group interactivity are encouraged. Participants frequently requested more discussion on returning to work and school, which is supported by previous research findings that returning to work or school is a common practical concern among AYA cancer survivors [16]. Additional details on plant-based proteins, managing fatigue from specific activities such as online meetings, and the social life of AYAs were requested by participants and should be considered in future iterations of CaRE-AYA.

Finally, the safety of the CaRE-AYA program was confirmed, as no serious adverse events occurred. This aligns with previous literature, including a review of exercise intervention studies for AYA patients, which concluded that these types of interventions are safe for this population [66].

Due to the small sample size and pilot nature of this study, the analysis of the secondary clinical outcomes remains exploratory. The results demonstrated no floor or ceiling effects and good variability, supporting their use in future AYA cancer rehabilitation programming. Wide confidence intervals were observed as a result of the small sample size but there were encouraging trends in all outcomes and these data will be useful in informing the design of future studies.

### Strengths and Limitations

This study adds important new information regarding the feasibility and acceptability of an innovative impairment-driven multidimensional cancer rehabilitation program and helps to fill an important gap in AYA survivorship care. A pragmatic approach ensures that the study identifies potential challenges, refines methods, and ultimately supports the design of a larger trial that is both feasible and generalizable to everyday clinical practice. The study provides a foundation for further refinement and large-scale testing of the CaRE-AYA program. The integration of qualitative methods provided an in-depth understanding of the feasibility and acceptability of the program and will be used to further refine and optimize the intervention. While the results are promising, it is important to interpret them in the context of the study’s limitations. Importantly, the study was conducted in a well-funded tertiary care center with established referral pathways to cancer rehabilitation programming. This limits the study’s generalizability, as smaller hospitals may face greater barriers in establishing an AYA-specific cancer rehabilitation program. The pragmatic approach can also lead to patient heterogeneity and higher non-adherence to treatment and missing data, although encouragingly, we found good adherence and low rates missing data in our study. Data saturation was assumed for the qualitative interviews; however, the study sample was not fully representative of the entire population. For instance, the sample consisted primarily of female and older AYAs (≥30 years old), which limited generalizability. However, for this trial, the results are hypothesized to be generalizable to other groups. This could be further tested by obtaining a more diverse sample in future research. Additionally, qualitative interviews were only conducted with the CaRE-AYA participants. Feedback from the RKins would provide a more comprehensive assessment of the program’s feasibility and acceptability and is recommended for future research. Finally, follow-up assessments were only conducted 3 months post-program. Therefore, the estimates of the long-term impact of the CaRE-AYA program are not available. An additional assessment six months post-program is recommended for future investigations.

## 5. Conclusions

The CaRE-AYA pilot study provides important insights into the feasibility, acceptability, and safety of a multicomponent cancer rehabilitation program for the AYA cancer population. While research highlighting the unique challenges and specific needs of AYA cancer survivors has increased [67], studies examining the development and effectiveness of cancer rehabilitation programs for AYA cancer survivors remain scarce [67]. The results from the current study provide important new information on AYA patient preferences regarding the design, format, and content of cancer rehabilitation programming, and offer suggestions for future program adaptations. Larger-scale studies are now required to provide evidence of the effectiveness of the CaRE-AYA program in improving disability, physical functioning, mental health, and social functioning in AYA cancer patients.

## Figures and Tables

**Table 1:** Demographics and medical characteristics of participants at baseline.

Variable	Total Participants (*n* = 25)
Age (years)	
Mean (SD)	32.8 (4.8)
Median (range)	34.0 (20–39)
Time since diagnosis (months)	
Mean (SD)	20.7 (19.0)
Median (range)	13.9 (3.1–84.3)
Diagnosis n (%)	
Hematological disease site	11 (44)
Breast disease site	8 (32)
Gynaecologic disease site	2 (8)
CNS disease site	1 (4)
Gastrointestinal disease site	1 (4)
Head and neck disease site	1 (4)
Sarcoma disease site	1 (4)
Sex n (%)	
Female	19 (76)
Male	6 (24)
Gender n (%)	
Female	18 (72)
Male	6 (24)
Prefer not to answer	1 (4)
On active treatment n (%)	
No	19 (76)
Yes	6 (24)
Treatment received n (%)	
Systemic treatment	21 (84)
Surgical treatment	13 (52)
Radiation therapy	13 (52)
Other	5 (20)
Ethnicity n (%)	
White/Caucasian	8 (32)
East Asian	4 (16)
South Asian	3 (12)
Other	3 (12)
Latin American	2 (8)
Southeast Asian	2 (8)
West Asian	2 (8)
Arab	1 (4)
Marital status n (%)	
Single, never married	14 (56)
Married/common law	10 (40)
Divorced/separated	1 (4)
Highest level of education completed n (%)	
Completed college/university	11 (44)
Post-graduate degree	5 (20)
Completed high school	3 (12)
Professional degree	3 (12)
Less than high school	1 (4)
Completed technical school	1 (4)
Other	1 (4)
Employment n (%) ^a^	
On disability/sick leave	8 (33)
Full time	6 (25)
Unemployed	4 (17)
Part-time	3 (13)
Other	3 (13)
Living arrangement n (%)	
Spouse/partner	12 (48)
Alone	6 (24)
Other family/friend	5 (20)
Other	2 (8)
Socioeconomic status n (%) ^b^	
Prefer not to answer	8 (33)
USD 40,000–$75,000	5 (21)
<USD 20,000	5 (21)
>USD 75,000	4 (17)
USD 20,000–USD 39,000	2 (8)
Reason for referral ^c^	
Deconditioning	15 (60)
Pain	12 (48)
Cancer-related fatigue	10 (40)
Limitations to mobility	9 (36)
Chemotherapy-induced peripheral neuropathy	7 (28)
Neurocognitive impairments	7 (28)
Lymphedema	3 (12)

Abbreviations: CNS, central nervous system; SD, standard deviation; n, sample size. ^a^ *n* = 24. ^b^ *n* = 24. ^c^ Patients presented with multiple impairments.

## Data Availability

The datasets presented in this article are not readily available because of REB requirements. Requests to access the datasets should be directed to the corresponding author (JMJ).

## Supplementary Materials

The following supporting information can be downloaded at https://www.mdpi.com/article/10.3390/cancers17071066/s1.
