# Supplementary material for: A Pilot Study to Evaluate the Feasibility and Acceptability of a Tailored Multicomponent Rehabilitation Program for Adolescent and Young Adult (AYA) Cancer Survivors
Source: Cancers (Basel). 2025 Mar 22;17(7):1066. doi: 10.3390/cancers17071066 (PMC11988022; doi:10.3390/cancers17071066)
Supplement: Supplementary file 1 [file cancers-17-01066-s001.zip › cancers-3453290-supplementary.pdf]

Supplemental File S1: Adaptations made to CaRE@ELLICSR for CaRE AYA

What was modified?	How was it modified?	Why was it modified?	Impact: Was the core function altered?
Intervention: Delivery – Group-based exercise classes	- Incorporated education on different forms of exercise (e.g., HIIT, super setting).<br>- Curation of a music playlist based on recommendations from group participants.	- Increase engagement and interest in exercise classes.<br>- Based on feedback from AYAs and instructors, and advisory workgroup discussions.	No
Intervention: Delivery – Self-management skills education classes	- Tailored class content to the AYA population (e.g., addition of FCR content).<br>- Increased interactive components in self-management skills education classes.	- Inclusion of topics relevant to this population as determined by the needs assessment survey results and advisory workgroup discussions.<br>- Increase engagement in education classes.	No
Intervention: Delivery – Hybrid format	- Hybrid education classes and in-person exercise classes.<br>- In-person attendance was still encouraged.	- Hybrid format requested on needs assessment survey by 69% of respondents (n=72).<br>- Delivery of hybrid education determined by expert advisory workgroup and CRS class instructors.	Yes, impacted access: increased access.
Intervention: Delivery – Resources	- Inclusion of AYA-specific resources (e.g., community resources).	- Based on advisory workgroup discussions	No

		and input from CRS class instructors.	
Intervention: Delivery – Evaluations	- Post-class evaluation surveys.<br>- Post-program evaluation surveys.	- Used as an acceptability outcome in the pilot study.	No

Abbreviations: AYA, adolescents and young adults; HIIT, high-intensity interval training; FCR, fear of cancer recurrence; CRS, cancer rehab and survivorship.

Supplemental File S2: Between group adaptations to the CaRE-AYA program

**What was modified?**	**How was it modified?**	**Why was it modified?**	**Impact: Was the core function altered?**
Intervention: Delivery – Program timing	- The CaRE-AYA program was rescheduled to the afternoon.	- Based on feedback from the participants in groups one and two and results from the need assessment survey	No
Intervention: Delivery – Education class content	- More discussion time in each self-management education class.<br>- The inclusion of at least one interactive component in each self-management education class.<br>- Inclusion of more return-to-work resources in the "Plan for Your Future" class.	- Based on feedback from the participants in groups one and two.	No
Intervention: Delivery – Virtual class resources	- Self-management education class resources and slides emailed to participants at the start of each week	- Based on feedback from group one participants.	No

Supplemental File S3: Qualitative Interview Results

Theme	Subtheme	Subtheme definition	Example
Program benefits experienced by CaRE-AYA patients	Acquirement of new knowledge and skills to manage disease impact	Participants found that the CaRE-AYA program was informative and provided them with strategies that were helpful in their cancer recovery.	"The topics that were covered were also very, you know informative, and like apropos to sort of what we need, to help us, you know get through" – Male lymphoma cancer survivor, aged 42
	Experienced improvement in cancer-related symptoms and overall health	Through the application of new strategies and consistent physical activity, participants found improvements in their cancer-related symptoms and overall health.	"I think by like I don't know maybe week five six I noticed things like before … I would never like run down the stairs. … if I did and I forgot I'd have to stop halfway and catch my breath. And I started to do that a bit more easily and … be like "oh that was different from like a week ago". – Female lymphoma cancer survivor, aged 37
	Obtained psychosocial support from the program	Participants emphasized the supportive nature of the CaRE-AYA program. The group-based aspect of the program enabled participants to connect with, relate to, and learn one another. Participants also felt supported by the program staff. Additionally, the class content and discussions supported participants and provided them with emotional validation.	"It's a weird age … and I really had no one to like, to make me feel like "okay where I'm at in life is perfectly normal". And then going through those sessions and some of those PowerPoints I brought home to my family and I showed them … I'm not so different from other people going through the same thing." – Female lymphoma cancer survivor, aged 30
	CaRE-AYA facilitated increased motivation to improve health behaviours	Participants expressed that the program gave them motivation and encouragement to exercise consistently, focus on their cancer recovery, and engage in healthy behaviours.	"Like those eight weeks helped me build this habit that I wasn't really consistently doing before and helped me build it in a way that was actually sustainable, which is different from what I had tried to [exercise] before." – Female lymphoma cancer survivor, aged 28

	Appreciated that the program was specifically for AYA cancer survivors	Participants articulated how beneficial it was that this program was specific to the AYA age group. They felt that this facilitated connections within the group as they could relate to the other participants. Participants appreciated the tailoring of the CaRE-AYA program content to the AYA age group. They felt that all the included topics were applicable, appropriate, and relatable for their age group.	"To contrast in like the other groups … most of the other participants would be talking about like I don't know like their life in retirement … I'm sure it's really great for them to be able to talk about that and relate to each other, but I felt kind of alone and couldn't relate to any of them in there" – Female lymphoma cancer survivor, aged 28
Facilitators of the CaRE-AYA program benefits	Provided access to helpful resources	The participants appreciated being provided with the slides and handouts so they could digest the information on their own time, share it with friends and family, and investigate additional resources and material. Participants were also provided with information on community and hospital-based programs and instructed on how to access individual support. Participants enjoyed having access to the wearable activity trackers and Physitrack™ as they felt it held them accountable, contributed to their motivation and creation of an exercise routine, and helped them track their progress.	"It was really nice that they provided you with the resources to so that you could kinda go on your own time and like look into certain things." – Female lymphoma cancer survivor, aged 20<br>"The fact that I had a FitBit throughout the week, it kept me, on track, and just helped me track my exercises so that was good. For example, if I wanted to focus on keeping my heart rate above 110 during the exercise I think, that was really good." – Male sarcoma cancer survivor, aged 26
	Program design was effective	Participants believed that the program design was effective. They appreciated the inclusion of both self-management skills	"I could say that I definitely enjoyed it, for sure. I thought it was really well structured and the fact that we had both like the exercise portion and then the education piece." – Female

		education and group-based exercise.	lymphoma cancer survivor, aged 20
	No concerns with the study requirements	The participant had no issues with the assessments (questionnaires, physiological assessments). They felt the questions asked were appropriate and applicable. They did not feel like the assessments were too long, invasive, or unsafe.	"I think it was applicable and appropriate. There wasn't like a question where I was like "I'm not answering that"." – Female lymphoma cancer survivor, aged 38
Identified drawbacks of the CaRE-AYA program	Varying information needs among group participants	Discussion on how the participant did not feel that all the topics were applicable to their experience or that they were already aware of the information. Participants also shared that they would have liked to have focused more on certain topics.	"Some things like brain fog having gone through it I realized I did have some effects but I think in some cases it might have been a little less than others because I didn't have the chemotherapy … but its still helpful." – Female breast cancer survivor, aged 38
	Program format could be improved	Participants had a few suggestions for the program delivery. Most frequently, participants requested more time for group discussion in the self-management skills education classes and an increase in the interactivity of the classes.	"I don't know how you would structure this but maybe like a designated 15 minutes out of every session, … just hearing everyone's experiences or what they think about something, I think like in a way it really helps." – Male sarcoma cancer survivor, aged 26
Barriers to program attendance and participation faced by CaRE-AYA participants	Less optimal program timing	Participants from groups with early morning classes emphasized that this was too early for them and that they would have preferred a later class time. In response, the remaining two CaRE-AYA groups were delivered in the afternoon, and better feedback was received.	"Like waking up in the morning was kind of difficult like I managed to do it … but I feel like if I lived a bit … further away I might find it more difficult." – Female lymphoma cancer survivor, aged 28
	Difficulty with transportation to CaRE-AYA	Many participants reported city traffic to be a significant barrier for them. Since the first two groups began early	"The timing of it, like the traffic was crazy, so it was like three hours to get there so that was a

		in the morning, participants found themselves commuting during Toronto's rush hour traffic, therefore increasing their travel time to the program.	bit difficult" – Female lymphoma cancer survivor, aged 20
	Symptom burden and illness affected participation	Symptom burden was a barrier for many participants and affected their ability to engage in physical activity. Illness unrelated to cancer was another barrier to program attendance resulting in some participants' absence.	"I had another follow-up biopsy to, like right at the end, that seemed to like limit my, exercise, … but they were accommodating and helped me you know figure out other things that I could do and to not, not strain that side." – Female breast cancer survivor, aged 38
	Underutilization of program tools	Although participants were provided access to a FitBit™, some participants stated that they did not utilize this tool.	"The FitBit (laughs) I wore it like twice. And I wore it at like home and when I went on walks and stuff like that and then I just didn't like how it felt. … I'm a little bit weird with things on my wrist so I didn't like it (laughs). … Once the battery died I didn't charge it. I just didn't like it." – Female lymphoma cancer survivor, aged 38
	Wished for increased visibility to AYA cancer survivors	During the qualitative interviews, a couple of participants suggested that this program should aim for increased visibility among AYAs. The youngest AYA participant expressed that, while they could still relate to other participants, they wished there were more peers closer to their age.	"So like the fact that we have this I feel like a lot of young people don't – they wouldn't be the ones like they wouldn't be actively looking for it. Whereas … like even people in their thirties or forties …that get cancer might be like "okay I need to do this, like I want to reach out, I want to see what's available." – Female lymphoma cancer survivor, aged 20

Supplemental File S4: Summary statistics overtime for each outcome

Outcomes	T0	T1	T2
WHODAS			
Mean (sd)	13.9 (8.1)	13.7 (8.8)	12.9 (7.2)
Median (min, max)	13 (0, 28)	9.5 (3.0, 32.0)	10 (5, 31)
SF-36 Physical Functioning			
Mean (sd)	66.2 (18.0)	70.2 (23.4)	69.7 (22.0)
Median (min, max)	60 (40, 100)	72.5 (30.0, 100.0)	75 (15, 100)
SF-36 Physical Component			
Mean (sd)	41.0 (7.7)	42.4 (9.3)	42.0 (8.7)
Median (min, max)	39.2 (28.0, 54.9)	41.7 (25.6, 60.9)	42.9 (25.4, 54.9)
SDI Overall			
Mean (sd)	15.9 (8.6)	16.4 (8.7)	13.6 (9.9)
Median (min, max)	14 (0, 32)	15.5 (4.0, 34.0)	12 (2, 37)
GAD-7			
Mean (sd)	8.9 (5.7)	7.5 (4.1)	7.6 (4.7)
Median (min, max)	7 (0, 20)	7.5 (1.0, 16.0)	8 (1, 16)
GSLTPAQ			
Mean (sd)	21.9 (15.0)	34.7 (20.9)	33.3 (22.9)
Median (min, max)	19 (0, 55)	34 (3, 83)	28.2 (6.0, 92.0)
6MWT (m)			
Mean (sd)	494.3 (97.7)	521.0 (89.9)	518.1 (80.1)
Median (min, max)	525.5 (210.0, 610.0)	539 (335, 670)	523.5 (357.0, 656.0)
Combined Grip Strength (kg)			
Mean (sd)	53.7 (13.5)	57.1 (16.0)	57.6 (14.0)
Median (min, max)	53 (35, 84)	55 (27, 94)	56 (34, 83)
